# Common statistical and research design problems in manuscripts submitted to high-impact medical journals

**DOI:** 10.1186/1756-0500-4-304

**Published:** 2011-08-19

**Authors:** Sara Fernandes-Taylor, Jenny K Hyun, Rachelle N Reeder, Alex HS Harris

**Affiliations:** 1 Center for Health Care Evaluation, VA Palo Alto Health Care System and Stanford University School of Medicine, 795 Willow Road (MPD-152), Menlo Park, CA 94025, USA; 2 National Center for PTSD, VA Palo Alto Health Care System, 795 Willow Road, Menlo Park, CA 94025, USA

## Abstract

**Background:**

To assist educators and researchers in improving the quality of medical research, we surveyed the editors and statistical reviewers of high-impact medical journals to ascertain the most frequent and critical statistical errors in submitted manuscripts.

**Findings:**

The Editors-in-Chief and statistical reviewers of the 38 medical journals with the highest impact factor in the 2007 Science Journal Citation Report and the 2007 Social Science Journal Citation Report were invited to complete an online survey about the statistical and design problems they most frequently found in manuscripts. Content analysis of the responses identified major issues. Editors and statistical reviewers (n = 25) from 20 journals responded. Respondents described problems that we classified into two, broad themes: A. statistical and sampling issues and B. inadequate reporting clarity or completeness. Problems included in the first theme were (1) inappropriate or incomplete analysis, including violations of model assumptions and analysis errors, (2) uninformed use of propensity scores, (3) failing to account for clustering in data analysis, (4) improperly addressing missing data, and (5) power/sample size concerns. Issues subsumed under the second theme were (1) Inadequate description of the methods and analysis and (2) Misstatement of results, including undue emphasis on p-values and incorrect inferences and interpretations.

**Conclusions:**

The scientific quality of submitted manuscripts would increase if researchers addressed these common design, analytical, and reporting issues. Improving the application and presentation of quantitative methods in scholarly manuscripts is essential to advancing medical research.

## Findings

Attention to statistical quality in medical research has increased in recent years owing to the greater complexity of statistics in medicine and the focus on evidence-based practice. The editors and statistical reviewers of medical journals are charged with evaluating the scientific merit of submitted manuscripts, often requiring authors to conduct further analysis or content revisions to ensure the transparency and appropriate interpretation of results. Still, many manuscripts are rejected because of irreparable design flaws or inappropriate analytical strategies. As a result, researchers undertake the long and arduous process of submitting to decreasingly selective journals until the manuscript is eventually published. Aside from padding the authors' résumés, publishing results of dubious validity benefits few and makes development of clinical practice guidelines more time-consuming [1,2]. This undesirable state of affairs might often be prevented by seeking statistical and methodological expertise [3] during the design and conduct of research and during data analysis and manuscript preparation.

To assist educators and medical researchers in improving the quality of medical research, we conducted a survey of the editors and statistical reviewers of high-impact medical journals to identify the most frequent and critical statistical and design-related errors in submitted manuscripts. Methods experts have documented the use and misuse of quantitative methods in medical research, including statistical errors in published works and how authors use analytical expertise in manuscript preparation [3-11]. However, this is the first multi-journal survey of medical journal editors regarding the problems they see most often and what they would like to communicate to researchers. Scientists may be able to use the results of this study as a springboard to improve the impact of their research, their teaching of medical statistics, and their publication record.

### Sample and Procedure

We identified the 20 medical journals from the "Medicine, General & Internal" and "Biomedical" categories with the highest impact factor in each of the 2007 Science Journal Citation Report and the 2007 Social Science Journal Citation Report. Journals that do not publish results with statistical analysis were discarded, yielding 38 high impact journals. Twelve of these journals endorse the CONSORT criteria for randomized controlled trials, 6 endorse the STROBE guidelines for observational studies, and 5 endorse PRISMA criteria for systematic reviews. These journals are listed in Additional file 1[12].

The Editors-in-Chief and identifiable statistical reviewers of these journals were mailed a letter informing them of the online survey and describing the forthcoming email invitation that contained an electronic link to the survey instrument (sent within the week). We sent one email reminder a week after the initial email invitation in spring of 2008. We also requested that the Editors-in-Chief forward the invitation to their statistically-oriented editors or reviewers in addition to or instead of completing the survey themselves. An electronic consent form with the principal investigator's contact information was provided to potential respondents emphasizing the voluntary and confidential nature of participation. The Stanford University Panel on Human Subjects approved the protocol. This is one in a series of five studies surveying the editors and reviewers of high-impact journals in health and social science disciplines (medicine, public health, psychology, psychiatry, and health services) [13,14].

### Survey Content

The survey contained three parts: (1) Short-answer questions about the journals for which the respondents served, how many manuscripts they handled in a typical month, and their areas of statistical and/or research design expertise; (2) The main, open-ended question which asked: "As an editor-in-chief or a statistically-oriented reviewer, you provide important statistical guidance to many researchers on a manuscript-by-manuscript basis. If you could communicate en masse to researchers in your field, what would you say are the most important (common and high impact) statistical issues you encounter in reviewing manuscripts? Please describe the issues as well as what you consider to be adequate and inadequate strategies for addressing them."; and (3) One to four follow-up questions based on the respondents' self-identified primary area of statistical expertise. These questions were developed by polling 69 researchers regarding what statistical questions they would want to ask the editors or statistical reviewers of major journals.

### Analysis

Responses to the open-ended questions were analyzed qualitatively using content analysis to identify dominant themes. We coded the responses to the main question on the most common and high impact (per the wording of the question) statistical issue and the respondents' proposed solutions to those issues. In the analysis phase, two of the authors resolved coding criteria and sorted the responses according to the two major categories that emerged from the data.

A. Statistical and sampling issues

B. Inadequate reporting clarity or completeness

The results are presented in each category from most frequently mentioned to least frequently mentioned.

### Respondent Characteristics

Respondents to the survey were comprised of 25 editors and statistical reviewers (of 60 solicited) who manage manuscripts from 20 of the 38 journals in the sampling frame. Respondents indicated reviewing or consulting on a mean of 47 (range: 0.5 to 250) manuscripts per month. The most frequently reported areas of expertise (multiple responses possible) were the design and analysis of clinical trials (n = 12), general statistics (n = 14), quasi-experimental/observational studies (n = 12), and epidemiology (n = 11).

### Respondents' Suggestions for Statistical and Sampling Issues

Respondents often noted problems that are fundamental to research design and quantitative methods, including analytical strategies that are incomplete or mismatched with the data structure or scientific questions, failure to address missing data, and low power. Below, we describe the specific issues mentioned by respondents and provide accessible references for more detailed discussion.

(1) Inappropriate or incomplete analysis: In addition to minor arithmetic and calculation errors, respondents expressed concern over researchers' choice of statistical tests. Specifically, frequent problems exist in the appropriateness of statistical tests chosen for the questions of interest and for the data structure. These include using parametric statistical tests when the sample size is small or in the presence of obviously violated assumptions [15]. In addition, researchers may fail to account for the sampling framework in survey-based studies with appropriate weighting of observations [16,17]. Other errors include confusing the exposure and outcome variables in the analysis phase. That is, in laboratory data, the exposure of interest is mistakenly analyzed as the outcome in analyses. In a similar vein, researchers sometimes mistakenly report the discrimination of a clinical prediction rule or internal validation method (e.g., bootstrap) using the training dataset rather than the test set [18,19]. Other concerns included creating dichotomous variables out of continuous ones without legitimate justification, thereby discarding information, and the use of stepwise regression analysis, which, among other problems, introduces bias into parameter estimates and tends to over-fit the data. See Malek, et al. [20] for a pithy discussion of the pitfalls of stepwise regression and additional references.

(2) The substantive area of analysis that received the most attention from respondents was the failure to account for clustered data and the use of hierarchical or mixed linear models. The reviewers often observed that authors fail to account for clustering when it is present. Examples of this include data collected on patients over time, where successive observations are dependent upon those in the previous time period(s), or multiple observations are nested in larger units (e.g., patients within hospitals). In these situations, reviewers prefer to see an analytical approach that does not have an independence assumption and properly accounts for clustering, including time series analysis, generalized linear mixed models, or generalized estimating equations where the population-averaged effect is of interest [21-24].

(3) Addressing missing data: Frequently, researchers fail to mention the missing data in their sample or fail to describe the extent of the missing data. Problems with low response rates in studies are often not addressed or are inadequately discussed. In addition, longitudinal studies may fail to address differential dropout rates between groups that may have an effect on the outcome. In addition, those researchers who do discuss missing data often do not describe their methods of data imputation or their evaluation of whether missing data are significantly related to any observed variables. Those researchers who do explicitly address missing data regularly use suboptimal approaches. For example, investigators with longitudinal data often employ complete case analysis, last observation carried forward (LOCF) or other single imputation methods. These approaches can bias estimates and understate the sample variance. Preferably, researchers would evaluate the missing at random (MAR) assumption and conduct additional sensitivity analyses if the MAR assumption is suspect [25,26]. In addition, a detailed qualitative description of the loss process is essential, including the likelihood of MAR and the likely direction of any bias.

(4) Power and sample size issues: Power was another area that reviewers mentioned as problematic. Respondents also noted that power calculations are not done at all or are done post hoc rather than being incorporated into the design and sampling framework [27]. In novel studies where no basis for power calculations exists, this should be explicitly noted.

(5) Researchers often use propensity scores without recognition of the potential bias caused by unmeasured confounding [28-30]. Propensity scores are the probabilities of the individuals in a study being assigned to a particular condition given a set of known covariates and are used to reduce the confounding of covariates in observational studies. The bias problem arises when an essential confounder is not measured, and the use of propensity scores in this situation can exacerbate the bias already present in an analysis.

### Respondents' Suggestions for Inadequate Reporting Clarity or Completeness

In addition to specific analytical concerns, respondents also reported common errors in the text of methods and results sections. Although some of these problems are semantic, others reflect a misinterpretation or misunderstanding of the methods employed.

(1) Inadequate description of methods and analysis: Respondents observed that manuscripts often do not contain a clear description of the analysis. Authors should provide as much methodological detail as possible, including targeted references and a statistical appendix if appropriate. One respondent provided a rule of thumb whereby an independent reader should be able to perform the same analysis based solely on the paper. Other issues included inadequate description of the study cohort, recruitment, and response rate, and the presentation of relative differences (e.g., odds ratio = 1.30) in the absence of absolute differences (e.g., 2.6% versus 2%). As one respondent wrote, "Since basic errors that are easily identified remain common, there is real concern of the presentation of analyses for more complex methods where the errors will not be testable by the reviewer."

(2) Miscommunication of results: Researchers frequently report likelihood ratios for diagnostic tests (the likelihood of an individual having a particular condition relative to the likelihood of an individual not having that condition given a certain test result) without associated sensitivity and specificity. Although this is very useful for learning how well a test of interest predicts the risk of a given result [31,32], editors also appreciate the inclusion of rates of true positives and true negatives to give the reader a complete picture of the analysis.

Respondents also noted an undue emphasis on p-values and excessive focus on significant results. For example, authors often highlight the significance of a categorical dummy that is not significant overall; the overall significance of a multi-category predictor should be tested by using an appropriate joint test of significance [33]. In turn, non-significant results are seldom presented in manuscripts. Authors leave out indeterminate test results when describing diagnostic test performance and fail to report confidence intervals along with p-values. An analogous problem is the "unthinking acceptance" of p < 0.05 as significant. Researchers can fall prey to alpha errors and take the customary but curious position of touting significance just below p < 0.05 and non-significance just above the 0.05 threshold. In addition, authors may trumpet a significant result in a large study when the size of the difference is clinically unimportant. In this situation, a focus on the effect size could be more appropriate [34].

## Discussion

Journal editors and statistical reviewers of high-impact medical journals identified several common problems that significantly and frequently affect the quality of submitted manuscripts. The majority of respondents underscored the fundamentals of research methods that should be familiar to all scientists. These include rigorous descriptions of sampling and analytic strategies, recognition of the strengths and drawbacks of a particular analytical approach, and the appropriate handling of missing data. Respondents also discussed concerns about more advanced methods in the medical research toolkit. Specifically, authors may not understand or report the limitations of their analysis strategies and hedge these with sensitivity analyses and more tempered interpretations. Finally, respondents emphasized the importance of the clear and accurate presentation of methods and results.

Although this study was not intended as a systematic or comprehensive catalog of all statistical problems in medical research, it does shed some light on common issues that delay or preclude the publication of research that might otherwise be sound and important. Moreover, the references included in this paper may provide some useful analytical guidance for researchers and for educators. Accordingly, this work serves to inform medical education and research to improve the overall quality of manuscripts and published research and to increase likelihood of publication.

In addition, these data provide evidence for the importance of soup-to-nuts methodological guidance in the research process. Statisticians and methodological experts should be consulted during the study design, analysis, and manuscript writing phases to improve the quality of research and to ensure the clear and appropriate application of quantitative methods. Although this may seem obvious, previous work by Altman and his colleagues demonstrates that this is rarely the case in medical research [3]. Rather, statistical experts are often consulted only during the analysis phase, if at all, and even then may not be credited with authorship [35]. In addition to statistical guidance, researchers should consult reporting guidelines associated with their intended research design, such as CONSORT for randomized, controlled trials, STROBE for observational studies, and PRISMA for systematic reviews. Adherence to such guidelines helps to ensure a common standard for reporting and a critical level of transparency in medical research. Professional organizations and prominent journals, including the Cochrane Collaboration and The Lancet, peer-review research protocols, which also helps to create a standard for research design and methods.

This work should be interpreted in light of several important limitations. We did not collect data on the professional position (e.g., academic department, industry, etc.) of the respondents and consequently do not know the composition of the sample or how this may have shaped our findings. Although the response rate was similar to other surveys of journal editors, and we have no reason to suspect significant response bias, the possibility of response bias remains. In addition, the size of our sample may limit the generalizability of our findings

Overall, this work is intended to inform researchers and educators on the most common pitfalls in quantitative medical research, pitfalls that journal editors note as problematic. Given the recent clinical research priorities of health care agenda-setting organizations, such as comparative effectiveness research and evidence-based practice, medical research is expected to meet a new bar in terms of valid and transparent inquiry [36-39]. Improving the application and presentation of quantitative methods in scholarly manuscripts is essential to meeting the current and future goals of medical research.

## Competing interests

The authors declare that they have no competing interests.

## Authors' contributions

SFT was responsible for the analysis and interpretation of data, drafting the manuscript, and final approval of the draft. JKH made substantial contributions to the conception and design of the study and the survey instrument, was involved in revising the manuscript, and gave final approval. RNR aided in data collection, analysis and interpretation of the data, manuscript revisions, and gave final approval. AHSH made substantial contributions to the conception and design of the study and the survey instrument, was involved in revising the manuscript, and gave final approval.

## Supplementary Material

Additional file 1 **Appendix**. 2007 Journal Citation Report titles included in the sampling frame. Click here for file

## References

[B1] Steinberg EP Luce BR Evidence Based? Caveat Emptor! Health Affairs 2005 24 1 80 92 10.1377/hlthaff.24.1.80 15647218

[B2] GRADE Working Group Grading quality of evidence and strength of recommendations BMJ 2004 1490 1493 10.1136/bmj.328.7454.1490 PMC428525 15205295

[B3] Altman DG Goodman SN Schroter S How statistical expertise is used in medical research JAMA 2002 287 21 2817 2820 10.1001/jama.287.21.2817 12038922

[B4] Altman DG Poor-quality medical research: what can journals do? JAMA 2002 287 21 2765 2767 10.1001/jama.287.21.2765 12038906

[B5] Chalmers I Altman D How can medical journals help prevent poor medical research? Some opportunities presented by electronic publishing Lancet 1999 353 9151 490 493 10.1016/S0140-6736(98)07618-1 9989737

[B6] Gardner M Bond J An exploratory study of statistical assessment of papers published in the British Medical Journal JAMA 1990 263 10 1355 10.1001/jama.263.10.1355 2304214

[B7] Goodman S Altman D George S Statistical Reviewing Policies of Medical Journals Caveat Lector? Journal of general internal medicine 1998 13 11 753 756 10.1046/j.1525-1497.1998.00227.x 9824521 PMC1497035

[B8] Gore S Jones G Thompson S The Lancet's statistical review process: areas for improvement by authors The Lancet 1992 340 8811 100 102 10.1016/0140-6736(92)90409-V 1351973

[B9] McKinney W Young M Hartz A Bi-Fong Lee M The inexact use of Fisher's exact test in six major medical journals JAMA 1989 261 23 3430 10.1001/jama.261.23.3430 2724487

[B10] Porter A Misuse of correlation and regression in three medical journals Journal of the Royal Society of Medicine 1999 92 3 123 10396255 10.1177/014107689909200306 PMC1297101

[B11] Schriger DL Altman DG Inadequate post-publication review of medical research BMJ 341 c3803 10.1136/bmj.c3803 20702543

[B12] Institute for Scientific Information Journal Citation Report 2007 Thompson Scientific

[B13] Harris AHS Reeder R Hyun JK Common statistical and research design problems in manuscripts submitted to high-impact psychiatry journals: What editors and reviewers want authors to know Journal of Psychiatric Research 2009 43 1231 1234 10.1016/j.jpsychires.2009.04.007 19435635

[B14] Harris AHS Reeder RN Hyun JK Common statistical and research design problems in manuscripts submitted to high-impact public health journals The Open Public Health Journal 2009 2 44 48 10.2174/1874944500902010044

[B15] Sheskin DJ Handbook of Parametric and Nonparametric Statistical Procedures 2007 4 Boca Raton: Chapman & Hall

[B16] Korn EL Graubard BI Analysis of large health surveys: Accounting for the sampling design Journal of the Royal Statistical Society Series A (Statistics in Society) 1995 158 2 263 295 10.2307/2983292

[B17] Lee ES Forthofer RN Eds Analyzing Complex Survey Data 2006 2 Thousand Oaks: Sage Publications, Inc

[B18] Browne MW Cross-validation methods Journal of Mathematical Psychology 2000 44 1 108 132 10.1006/jmps.1999.1279 10733860

[B19] Efron B Gong G A leisurely look at the bootstrap, the jackknife, and cross-validation The American Statistician 1983 37 1 36 48 10.2307/2685844

[B20] Malek MH Berger DE Coburn JW On the inappropriateness of stepwise regression analysis for model building and testing European Journal of Applied Physiology 2007 101 263 264 10.1007/s00421-007-0485-9 17520270

[B21] Diggle PJ Heagerty PJ Liang KY Zeger SL Analysis of Longitudinal Data 2002 New York: Oxford University Press

[B22] Hardin JW Hilbe JM Generalized Estimating Equations 2003 Boca Raton: Chapman & Hall

[B23] Raudenbush SW Bryck AS Hierarchical Linear Models: Applications and Data Analysis Methods 2002 2 Thousand Oaks: Sage Publications, Inc

[B24] Snijders T Bosker RJ Multilevel Analysis 1999 Thousand Oaks: Sage Publications, Inc

[B25] Daniels M Hogan J Missing Data in Longitudinal Studies: Strategies for Bayesian Modeling and Sensitivity Analysis 2008 New York: Chapman & Hall

[B26] Rubin DB Inference and missing data Biometrika 1978 63 581 592

[B27] Zumbo B Hubley A A note on misconceptions concerning prospective and retrospective power The Statistician 1998 47 2 385 388

[B28] D'Agostino RB Propensity score methods for bias reduction in the comparison of a treatment to a non-randomized control group Statistics in Medicine 1998 17 2265 2281 10.1002/(SICI)1097-0258(19981015)17:19<2265::AID-SIM918>3.0.CO;2-B 9802183

[B29] Luellen JK Stadish WR Clark MH Propensity scores: An introduction and experimental test Eval Rev 2005 29 6 530 558 10.1177/0193841X05275596 16244051

[B30] McCaffrey DF Ridgeway G Morral AR Propensity score estimation with boosted regression for evaluating causal effects in observational studies Psychological Methods 2004 9 4 403 425 15598095 10.1037/1082-989X.9.4.403

[B31] Altman DG Bland JM Diagnostic tests 1: Sensitivity and specificity BMJ 1994 308 1552 8019315 10.1136/bmj.308.6943.1552 PMC2540489

[B32] Deeks JJ Altman DG Diagnostic tests 4: Likelihood ratios BMJ 2004 329 168 169 10.1136/bmj.329.7458.168 15258077 PMC478236

[B33] Wooldridge JM Introductory Econometrics: A modern approach 2009 Mason, OH: South-western Cengage Learning

[B34] Gotzsche PC Believability of relative risks and odds ratios in abstracts: Cross sectional study BMJ 2006 333 7561 231 4 10.1136/bmj.38895.410451.79 16854948 PMC1523498

[B35] Bacchetti P Peer review of statistics in medical research: The other problem BMJ 2002 324 7348 1271 3 10.1136/bmj.324.7348.1271 12028986 PMC1123222

[B36] Evidence-based Medicine http://www.ahrq.gov/browse/evidmed.htm

[B37] Institute of Medicine The Learning Healthcare System: Workshop Summary (IOM Roundtable on Evidence-Based Medicine) 2007 Washington, DC: National Academies Press 21452449

[B38] Institute of Medicine Initial National Priorities for Comparative Effectiveness Research 2009 Washington, DC: National Academies Press

[B39] Lang TA Secic M How to Report Statistics in Medicine: Annotated guidelines for authors, editors, and reviewers 1997 Philadelphia: American College of Physicians

