# Supplementary material for: Common statistical and research design problems in manuscripts submitted to high-impact medical journals
Source: BMC Res Notes. 2011 Aug 19;4:304. doi: 10.1186/1756-0500-4-304 (PMC3224575; doi:10.1186/1756-0500-4-304)
Supplement: Additional file 1 — Appendix. 2007 Journal Citation Report titles included in the sampling frame. [file 1756-0500-4-304-S1.DOC]

| **APPENDIX: 2007 Journal Citation Report Titles Included in the Sampling Frame** | |
| --- | --- |
| **Journal Title** | **Category** |
| AIDS AND BEHAVIOR | Biomedical |
| AIDS CARE-PSYCHOLOGICAL AND SOCIO-MEDICAL ASPECTS OF AIDS/HIV | Biomedical |
| CULTURE, HEALTH & SEXUALITY | Biomedical |
| DEATH STUDIES | Biomedical |
| EVOLUTION AND HUMAN BEHAVIOR | Biomedical |
| HEALTH | Biomedical |
| HEALTH, RISK & SOCIETY | Biomedical |
| HUMAN NATURE-AN INTERDISCIPLINARY BIOSOCIAL PERSPECTIVE | Biomedical |
| INTERNATIONAL FAMILY PLANNING PERSPECTIVES | Biomedical |
| JOURNAL OF FAMILY PLANNING AND REPRODUCTIVE HEALTH CARE | Biomedical |
| JOURNAL OF HEALTH POLITICS, POLICY AND LAW | Biomedical |
| JOURNAL OF MEDICAL ETHICS | Biomedical |
| JOURNAL OF MEDICINE AND PHILOSOPY | Biomedical |
| MEDICAL ANTHROPOLOGY QUARTERLY | Biomedical |
| PSYCHO-ONCOLOGY | Biomedical |
| SOCIAL SCIENCE & MEDICINE | Biomedical |
| SOCIOLOGY OF HEALTH & ILLNESS | Biomedical |
| AMERICAN JOURNAL OF MEDICINE | Medicine, General and Internal |
| AMERICAN JOURNAL OF PREVENTIVE MEDICINE | Medicine, General and Internal |
| ANNALS OF FAMILY MEDICINE | Medicine, General and Internal |
| ANNALS OF INTERNAL MEDICINE | Medicine, General and Internal |
| ANNALS OF MEDICINE | Medicine, General and Internal |
| ANNUAL REVIEW OF MEDICINE | Medicine, General and Internal |
| ARCHIVES OF INTERNAL MEDICINE | Medicine, General and Internal |
| BRITISH MEDICAL JOURNAL | Medicine, General and Internal |
| CANADIAN MEDICAL ASSOCIATION JOURNAL | Medicine, General and Internal |
| CURRENT MEDICAL RESEARCH AND OPINION | Medicine, General and Internal |
| EUROPEAN JOURNAL OF CLINICAL INVESTIGATION | Medicine, General and Internal |
| JOURNAL OF GENERAL INTERNAL MEDICINE | Medicine, General and Internal |
| JOURNAL OF INTERNAL MEDICINE | Medicine, General and Internal |
| JAMA-JOURNAL OF THE AMERICAN MEDICAL ASSOCIATION | Medicine, General and Internal |
| LANCET | Medicine, General and Internal |
| MAYO CLINIC PROCEEDINGS | Medicine, General and Internal |
| MEDICAL CARE | Medicine, General and Internal |
| MEDICINE | Medicine, General and Internal |
| NEW ENGLAND JOURNAL OF MEDICINE | Medicine, General and Internal |
| PLOS MEDICINE | Medicine, General and Internal |
| QJM-AN INTERNATIONAL JOURNAL OF MEDICINE | Medicine, General and Internal |
